# Phage-Encoded Depolymerase DepKP144 with Therapeutic Potential Against Both K1- and K2-Type *Klebsiella pneumoniae*

**DOI:** 10.3390/ijms27125466

**Published:** 2026-06-17

**Authors:** Ekaterina A. Kondakova, Natalia N. Golosova, Bogdana I. Kravchuk, Yana A. Khlusevich, Vyacheslav I. Yakubovskij, Yuliya N. Kozlova, Svetlana A. Grishkova, Nina V. Tikunova, Andrey L. Matveev

**Affiliations:** 1 Institute of Chemical Biology and Fundamental Medicine Siberian Branch of Russian Academy of Sciences, 630090 Novosibirsk, Russia; e.kondakova1@g.nsu.ru (E.A.K.); n.golosova@alumni.nsu.ru (N.N.G.); semali328@gmail.com (B.I.K.); khlusevichjana@mail.ru (Y.A.K.); yakubovskij97@xmail.ru (V.I.Y.); ulona79@mail.ru (Y.N.K.); jelikk5@gmail.com (S.A.G.); tikunova@1bio.ru (N.V.T.); 2 Faculty of Natural Sciences, Novosibirsk State University, 630090 Novosibirsk, Russia

**Keywords:** *Klebsiella pneumoniae*, depolymerase, multi drug resistance, antibacterials, K1-type, K2-type, capsular polysaccharide, biofilm degradation, *K. pneumoniae*

## Abstract

Multidrug resistance (MDR) is a global problem for the healthcare system, complicating the therapy of bacterial infections. It is noted that patients infected with MDR strains often require prolonged hospitalization, have a high risk of mortality, and remain vulnerable to reinfection after recovery. In this study, recombinant phage-encoded depolymerase DepKP144 was produced using the *Escherichia coli* expression system and purified. The depolymerase DepKP144 protein was able to reduce viable bacterial counts following capsule degradation in 95% of the tested strains of type K1 and 85% of the tested strains of type K2 *Klebsiella pneumoniae*. The depolymerase DepKP144 was active against *K. pneumoniae* K1-type and K2-type planktonic cells and destroyed the biofilms formed by clinical MDR strains of *K. pneumoniae.* In in vivo experiments, DepKP144 at a dose of 180 μg/mouse resulted in a 50% survival of the mice infected with K2-type *K. pneumoniae* and in a 17% survival of the mice infected with K1-type *K. pneumoniae*. This depolymerase is promising for further development of prevention and therapeutic candidates against MDR *K. pneumoniae*.

## 1. Introduction

Multidrug resistance (MDR) is a global problem for the healthcare system, complicating the therapy of bacterial infections. It is noted that patients infected with MDR strains often require prolonged hospitalization and have a high risk of mortality. Also, after treatment, they can experience prolonged hospitalization, and after recovery, they remain vulnerable to re-infections [1]. In 2024, the World Health Organization (WHO) provided a list of 24 antibiotic-resistant bacterial pathogens for which new drugs need to be developed. These include *Klebsiella pneumoniae* resistant to carbapenems (CRKP) and third-generation cephalosporins. These bacteria are categorized as “critical” pathogens, i.e., the highest level of prioritization [2,3].

*Klebsiella* spp. is a genus of Gram-negative encapsulated immobile bacilliform bacteria belonging to the family *Enterobacteriaceae* of the order *Enterobacterales*. The genus includes more than 12 species, among which the most clinically significant are *K. pneumoniae* and *Klebsiella oxytoca* [4]. These are opportunistic pathogens of the group ESKAPE group, infesting the skin and mucous membranes of human organs and can cause immunocompromised diseases such as pneumonia, urinary tract infections, intestinal infections and others [5,6].

Some strains of *K. pneumoniae* belong to the “hypervirulent” pathotype (hvKp) and, unlike “classical” strains, have siderophore proteins (aerobactin, enterobactin) and allantoin ion utilization systems [7,8,9]. These pathogenicity factors favor metastatic spread of *Klebsiella* to distant organs and the development of invasive forms of infections (liver, brain, spleen abscess, meningitis, endophthalmitis, necrotizing fasciitis, subdural empyema) even in immunocompetent individuals [10,11,12]. A characteristic feature of most “hypervirulent” strains is the presence of rmpA/A2 genes associated with the “hypermucoid” phenotype (hmv) [13,14]. This phenotype is manifested by increased production of the capsular polysaccharides that mask cell-surface structures and protect the bacterium from host immune factors such as phagocytes and complement proteins. In addition, the capsule impedes the penetration of cationic antimicrobial peptides and antibiotics to their targets [15]. The most common serotypes with high virulence are K1 and K2, which account for about 70% of hvKp isolates [16]. Reports of cases of convergence of “hypervirulence” and MDR determinants are particularly alarming [17]. The evolution of *Klebsiella* resistance to the mainstream β-lactam antibiotics in therapy has led to the emergence and spread of multidrug-resistant strains, or “superbacteria”, producing extended-spectrum β-lactamases (ESBLs) and carbapenemases [18].

To overcome this resistance mechanism, the use of enzymes capable of degrading exopolysaccharides, such as depolymerases, is promising. Phage depolymerases are enzymes that can cleave O-glycosidic bonds in the exopolysaccharides of Gram-negative bacteria. Most often, depolymerizing activity is exhibited by tail fiber proteins and tail spike proteins. During phage adhesion, the depolymerase binds to polysaccharides on the bacterial cell surface and hydrolyzes them into soluble oligosaccharides, thereby facilitating viral access to specific cellular receptors [19].

Depending on the enzymatic activity, depolymerases are generally divided into two main groups: hydrolases (EC 3.2.1.) and lyases (EC 4.2.2.) [20]. Hydrolases cleave α-1,4-glycosidic bonds using a water molecule, and their active site is formed by pairs of aspartic or glutamic acid residues [21,22]. Lyases cleave the single bond between the monosaccharide and the C4 carbon in uronic acid via β-elimination, introducing a double bond between the C4 and C5 positions of the acid molecule without the involvement of water [21,23]. Most of the studied polysaccharide depolymerases are lyases, but some remain unclassified. They can inhibit biofilm formation, though their mechanism of action is not fully understood [24]. It is worth noting that polysaccharide depolymerases typically exhibit narrow specificity; however, their specific cleavage site can be present in various polysaccharides, allowing the enzymes to bind to multiple substrates [25].

In this study, the recombinant bacteriophage-encoded depolymerase DepKP144 from broadly lytic bacteriophage KlebP_144 was successfully expressed in the *E. coli* expression system and subsequently purified from inclusion bodies and refolded. The purified enzyme exhibited potent in vitro activity, degrading the capsule in 95% of tested *Klebsiella pneumoniae* capsular type K1 strains and 85% of type K2 strains in the spot-diffusion test. Furthermore, DepKP144 demonstrated weak lytic activity against both K1- and K2-type planktonic cells and effectively degraded preformed biofilms and inhibited biofilm formation MDR K1- and K2-type *K. pneumoniae* isolates. In vivo evaluation revealed that administration of DepKP144 at a dose of 180 μg per mouse led to protection in the murine abscess model, resulting in 50% survival in mice challenged with K2-type *K. pneumoniae* and 17% survival in those infected with K1-type strains. These results highlight the therapeutic potential of DepKP144 and support its further development as a promising candidate for the prevention and treatment of infections caused by MDR *K. pneumoniae*.

## 2. Results

### 2.1. In Silico Characterization of DepKP144

To determine the phylogenetic position of the full-length depolymerase DepKP144 among known phage-encoded homologs, a sequence-based analysis was performed using sequences retrieved from the NCBI GenBank database. Maximum likelihood tree reconstruction revealed that DepKP144 (PV528436) forms a well-supported clade (bootstrap 99) with depolymerases of *Klebsiella* phages Kp9, Kp11, *K. pneumoniae* phage strain 5899STDY8049220, and RCIP0048, clearly separated from the major heterogeneous cluster of *Klebsiella* phage depolymerases characterized by predominantly low internal bootstrap support (13–68). A distinct basal position was occupied by phages IME183 and Bacteriophage sp. isolate 3323 (bootstrap 99), representing the most divergent lineage. Notably, depolymerases from two *Raoultella* phages were interspersed among *Klebsiella* phage sequences, consistent with the known phylogenetic proximity of these genera.

**Figure 1:**
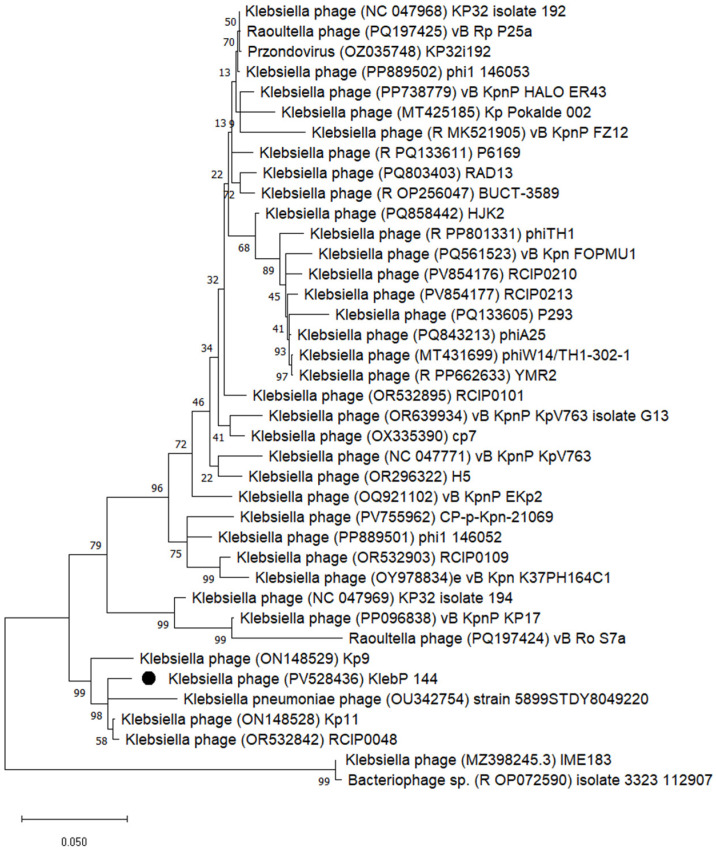
Phylogenetic analysis of the full-length depolymerase DepKP144 nucleotide sequences. The tree was constructed using the Maximum Likelihood method with the Kimura 2-parameter model (+G, +I) in MEGA X (1000 bootstrap replicates). Bootstrap support values are shown next to the branches. The filled circle (●) indicates KlebP 144 (PV528436), the phage used in this study. Scale bar: 0.050 substitutions per site.

The distinct positioning of DepKP144 outside the major cluster suggests independent evolutionary diversification, potentially reflecting adaptation to a specific subset of capsular polysaccharide structures (Figure 1).

**Figure 2:**
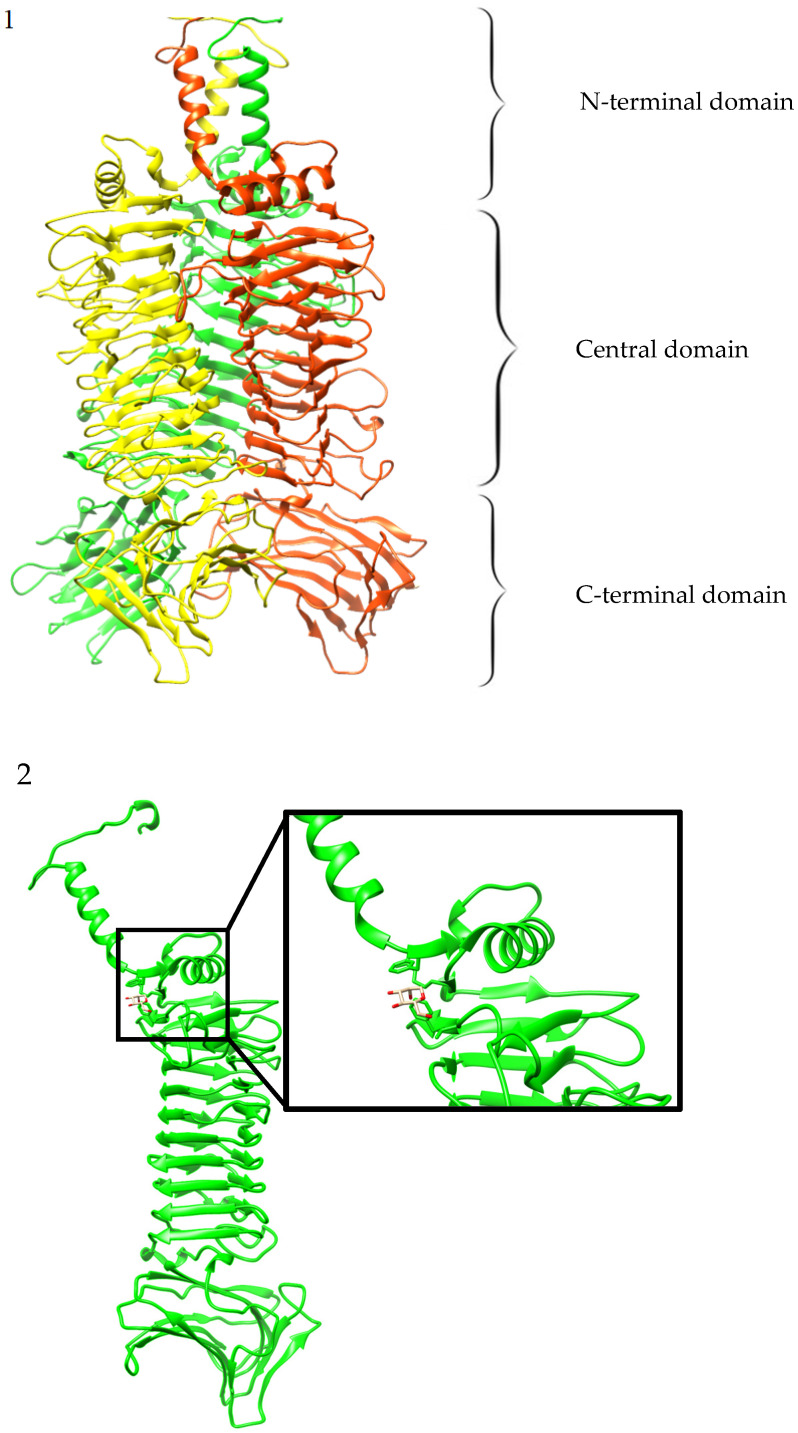
Predicted modular three-domain structure of the tail fibers of *Klebsiella* phage KlebP_144. (**1**) Side view: N-terminal domain required for attachment of the trimer to the tail structure of the virion; central domain with putative enzymatic activity; C-terminal domain responsible for protein trimerization and/or target recognition on the bacterial cell surface. The subunits of the DepKP144 homotrimer are colored red, yellow, and green. (**2**) Putative glycan-binding site of DepKP144 on the surface of the central domain. Amino acid residues Gly37, Thr58, Val67, and Asp86, which form the predicted catalytic site, are shown in stick representation. The three-dimensional structure of DepKP144 was predicted and rendered using UCSF Chimera, version 1.15.

The putative depolymerase DepKP144 is a homotrimer. The molecular weight of the monomer is approximately 56 kDa. The monomer of DepKP144 consists of 525 amino acid residues and has a theoretical isoelectric point (pI) of 9.21. The putative trimer structure of DepKP144 was predicted using AlphaFold2 and AlphaFold3 (https://alphafoldserver.com/, accessed on 20 January 2026). Domain analysis was performed using the HHPred tool (https://toolkit.tuebingen.mpg.de/tools/hhpred, accessed on 15 February 2026) and the InterProScan software package v.108 (https://www.ebi.ac.uk/interpro/search/sequence/, accessed on 15 February 2026). The analysis confirmed that the DepKP144 trimer consists of three domains. The N-terminal anchor domain (amino acids 1–34) is responsible for attachment to the bacteriophage. The central β-roll catalytic domain (amino acids 35–353) contains a core region with homology to both lyases and hydrolases. Using COFACTOR, it was predicted that a probable glycan-binding site is formed within this domain. The C-terminal jelly-roll domain is similar to the carbohydrate-binding module found in both lyases and hydrolases. Using COFACTOR, it was additionally predicted that a probable glycan-binding site is formed by Gly37, Thr58, Val67, and Asp86 (Figure 2).

The tail fiber protein DepKP144 belongs to a modular class of polysaccharide depolymerases with a typical three-domain architecture, comprising an N-terminal virion-anchoring domain, a central catalytic domain, and a C-terminal carbohydrate-binding/trimerization domain. This organization is characteristic of tailspike-like depolymerases that recognize and cleave capsular polysaccharides in *Klebsiella* and related genera. Unlike most previously described *Klebsiella* depolymerases, DepKP144 forms a separate, well-supported clade in the phylogenetic tree.

### 2.2. Cloning, Expression and Purification of Recombinant Depolymerase Protein DepKP144

The nucleotide sequence encoding the putative capsular depolymerase DepKP144 was amplified from the genomic DNA of the isolated *K. pneumoniae* bacteriophage and ligated into the T7 promoter-driven expression vector pET28a. The resulting construct, pET28_DepKP144, was introduced into *Escherichia coli* BL21 (DE3) cells to produce recombinant protein DepKP144. A range of cultivation parameters were screened in an effort to enhance protein solubility. Despite these efforts, the recombinant DepKP144 partitioned exclusively with the insoluble cellular fraction, forming inclusion bodies under all conditions evaluated (Figure S1).

The recombinant protein was subsequently isolated from inclusion bodies under denaturing conditions using immobilized metal affinity chromatography on Ni–NTA agarose. Following purification, DepKP144 was subjected to refolding, which was carried out during a stepwise dialysis procedure employing a descending urea gradient. SDS-PAGE analysis revealed that the purified protein migrated with an apparent molecular mass of approximately 56 kDa, in agreement with the theoretically predicted value. The presence of the C-terminal polyhistidine tag was verified by Western blotting with monoclonal anti-His antibodies (mAb.His1, Biosan, Russia). Densitometric evaluation of Coomassie-stained SDS-PAGE gels indicated that the purity of refolded DepKP144 reached approximately 90% (Figure S2).

The purification procedure yielded approximately 20 mg of recombinant DepKP144 per liter of bacterial culture. The purified protein was maintained at 4 °C in S-buffer (50 mM Tris–HCl, pH 7.5, 300 mM NaCl) at a concentration no lower than 1 mg/mL. Purified DepKP144 preparation obtained by Ni–NTA chromatography and refolding was used in all subsequent in vitro and in vivo assays.

### 2.3. Analysis of Enzymatic Activity of DepKP144 Against K. pneumoniae Using the Diffusion Assay

**Figure 3:**
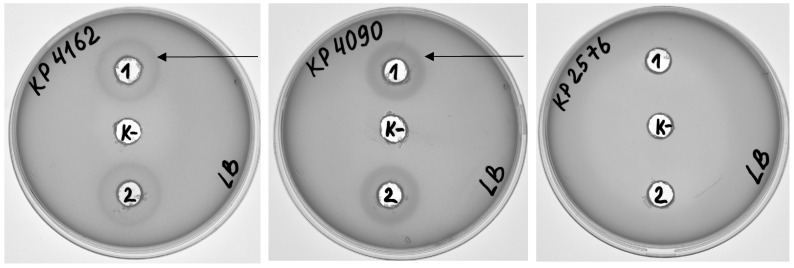
Hydrolytic activity of the DepKP144 protein against *K. pneumoniae* K2-type strains, determined using a diffusion test. 1–300 μg/mL of the recombinant protein DepKP144; 2–60 μg/mL of the recombinant protein DepKP144; K−—negative control R-buffer (50 mM Tris-HCl; pH 8.0). The halo around the wells (anti-capsular activity of the protein) is indicated by an arrow.

The enzymatic activity of DepKP144 was evaluated against a panel of clinical *K. pneumoniae* isolates sourced from the Culture Collection of Extremophilic Microorganisms and Type Cultures (CEMTC), ICBFM SB RAS, employing a diffusion-based assay. DepKP144 exhibited catalytic activity toward 30 out of 35 tested K2-type *K. pneumoniae* strains. In comparison, the parental bacteriophage KlebP_144 was capable of lysing only 19 of these 35 isolates (Table S2). The analyzed strain collection comprised both phage-susceptible and phage-resistant variants and included mucoid and hypermucoid morphotypes, as well as antibiotic-sensitive and MDR phenotypes (Figure 3, Table S1).

**Figure 4:**
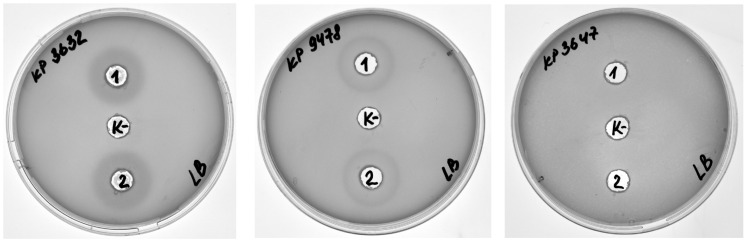
Hydrolytic activity of the DepKP144 protein against *K. pneumoniae* K1-type strains, determined using a diffusion test. 1–300 μg/mL of the recombinant protein DepKP144; 2–60 μg/mL of the recombinant protein DepKP144; K−—negative control R-buffer (50 mM Tris-HCl; pH 8.0). The halo around the wells (anti-capsular activity of the protein) is indicated by an arrow.

Applying the same methodological approach, the activity of DepKP144 was further examined against 19 clinical isolates of *K. pneumoniae* belonging to the K1 capsular serotype. It should be emphasized that the parental bacteriophage KlebP_144 is unable to infect K1-type strains. Notably, DepKP144 displayed unambiguous enzymatic activity toward 18 of the 19 K1 isolates tested (Figure 4, Tables S1 and S2).

**Figure 5:**
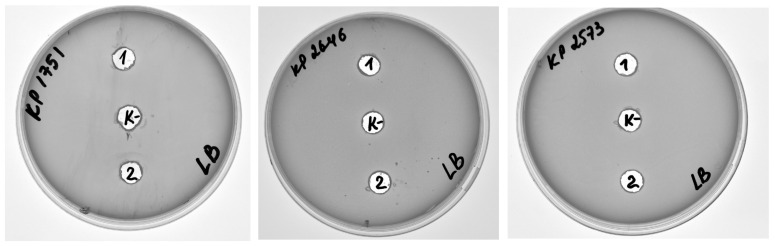
Hydrolytic activity of the DepKP144 protein against *K. pneumoniae* K9, K16, K17, K22, K35, K49, K51, K57, K63, and K108 strains, determined using a diffusion test. 1–300 μg/mL of the recombinant protein DepKP144; 2–60 μg/mL of the recombinant protein DepKP144; K−—negative control R-buffer (50 mM Tris-HCl; pH 8.0).

Prompted by the dual activity against K1 and K2 types, the substrate specificity of DepKP144 was subsequently probed against 10 isolates covering other capsular serotypes (K9, K16, K17, K22, K35, K49, K51, K57, K63, and K108). None of these strains were susceptible to enzymatic degradation (Figure 5, Table S1). Thus, DepKP144 exhibits narrow specificity, restricted to K1- and K2-type *K. pneumoniae*, with no measurable activity against the remaining serotypes examined.

### 2.4. Analysis of Anticapsular Activity of DepKP144 Against Planktonic Cells of K. pneumoniae In Vitro

**Figure 6:**
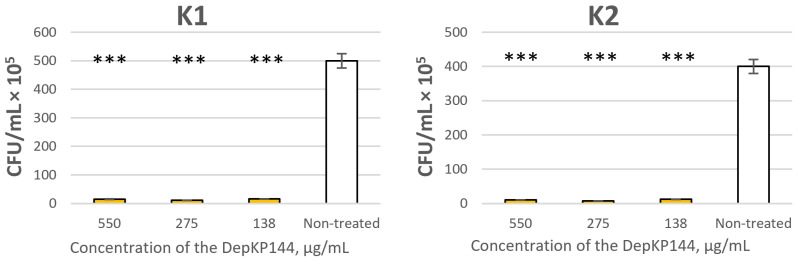
Anti-capsular activity of DepKP144 against *K. pneumoniae* strains CEMTC 9596 (K1-type) and 2067 (K2-type). DepKP144 was added in concentrations of 550, 275, and 138 µg/mL to *Klebsiella* cells and was incubated for two hours before viable colony counting. Cell cultures with R-buffer were used as controls. Experiments were performed in triplicate. *** *p* < 0.01.

The enzymatic activity of DepKP144 was quantified against *K. pneumoniae* strains CEMTC 9596 (K1-type) and 2067 (K2-type) using a serial dilution assay, as previously described. At a concentration of 550 µg/mL, DepKP144 reduced the viable count of the K1 strain by 45-fold, while concentrations of 275 µg/mL and 138 µg/mL achieved 33-fold and 31-fold reductions, respectively. Against the K2 strain, the corresponding reductions were 57-, 40-, and 33-fold at the same protein concentrations. These data indicate that DepKP144 exerts concentration-dependent anti-capsular activity toward both K1 and K2 capsular types of *K. pneumoniae*. At all concentrations tested (550, 275, and 138 µg/mL), the observed decreases in bacterial viability were statistically significant relative to the untreated controls (Figure 6).

### 2.5. Antibiofilm Activity of DepKP144 Against K. pneumoniae

**Figure 7:**
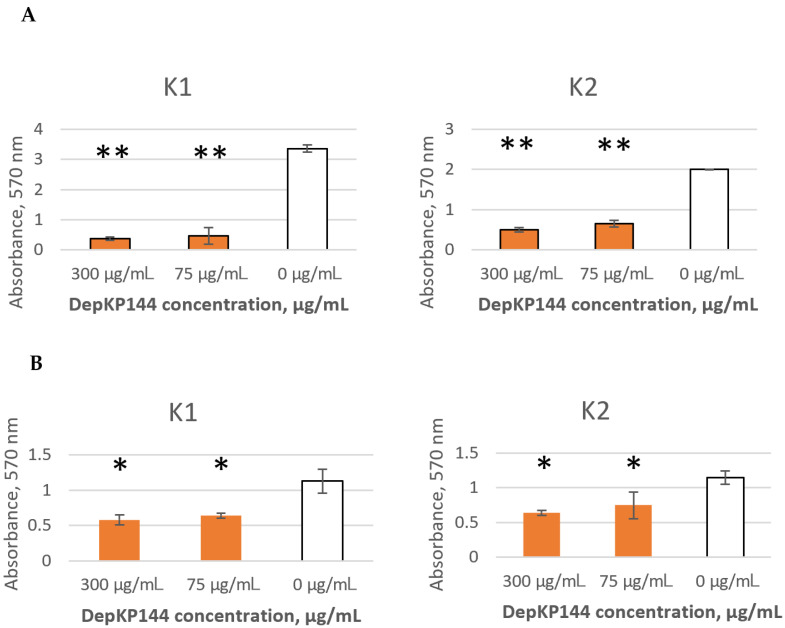
(**A**) Hydrolytic activity of DepKP144 against mature biofilms. (**B**) Inhibitory activity of DepKP144 on biofilm formation by the mucoid *K. pneumoniae strain* CEMTC 2574 (K2-type). DepKP144 was added at concentrations of 300 and 75 µg/mL to *K. pneumoniae* cells, followed by incubation for two days. Biofilms were then stained with crystal violet. Cells treated with R buffer served as controls. Experiments were performed in triplicate. * *p* < 0.05; ** *p* < 0.01.

The depolymerase DepKP144 exhibited strong hydrolytic activity against mature biofilms formed by both tested *K. pneumoniae* strains. Treatment of mature biofilms with 300 μg/mL DepKP144 led to a reduction in biofilm biomass of up to 89% for the K1-type biofilm and up to 75% for the K2-type biofilm. At a concentration of 75 μg/mL, DepKP144 treatment reduced biofilm biomass by up to 85% for the K1-type biofilm and up to 68% for the K2-type biofilm (Figure 7A). The obtained results show that the depolymerase DepKP144 is capable of degrading biofilms formed by *K. pneumoniae* of both K1 and K2 types.

Treatment with the depolymerase DepKP144 affected biofilm formation in a strain-dependent manner. Notably, a reproducible reduction in total biofilm formation was observed for both *K. pneumoniae* strain CEMTC 2894 (K1-type) and *K. pneumoniae* strain CEMTC 2574 (K2-type). At a concentration of 300 μg/mL, DepKP144 decreased biofilm formation by approximately 45–50% for both tested strains, whereas a lower concentration of 75 μg/mL resulted in a reduction of about 55–65% (Figure 7B).

### 2.6. In Vivo Evaluation of DepKP144 Depolymerase Activity in a Murine Klebsiella Infection Model

The anti-capsular efficacy of DepKP144 was evaluated in vivo using a murine model of systemic *Klebsiella* infection. Mice were randomly assigned to the following treatment groups: (i) intact controls (uninfected and untreated); (ii) protein controls (uninfected, treated with DepKP144); (iii) infection controls (infected, untreated); (iv) negative protein controls (infected, treated with 20 µg or 100 µg of the heterologous protein NS1 per animal); (v) phage-treated group (infected, treated with 10^9^ PFU of bacteriophage KlebP_144 per mouse); and (vi) DepKP144-treated groups (infected, receiving either 20 µg or 180 µg of DepKP144 alone per mouse).

During the first 72 h post-infection, all infected animals–regardless of treatment–displayed pronounced clinical signs of systemic infection, including reduced food intake, dehydration, hypertonia, and a body weight loss of approximately 1–2 g relative to baseline. The sole exception was the phage-treated group infected with *K. pneumoniae* strain CEMTC 2067 (capsular serotype K2), in which body weight recovery began as early as day two post-infection. From day four onward, surviving animals in the other experimental groups gradually resumed normal activity and showed a net weight gain of 2–3 g above their initial values.

All mice in the negative protein control groups (NS1) reached humane endpoints within 24–48 h post-infection, confirming that the unrelated protein elicited no nonspecific protective effects. In contrast, animals in the intact control group remained clinically healthy throughout the observation period, showing steady weight gain and normal behavior. Microbiological examination of tissue homogenates from surviving mice across all experimental and control groups yielded no culturable bacteria, indicating complete pathogen clearance.

**Figure 8:**
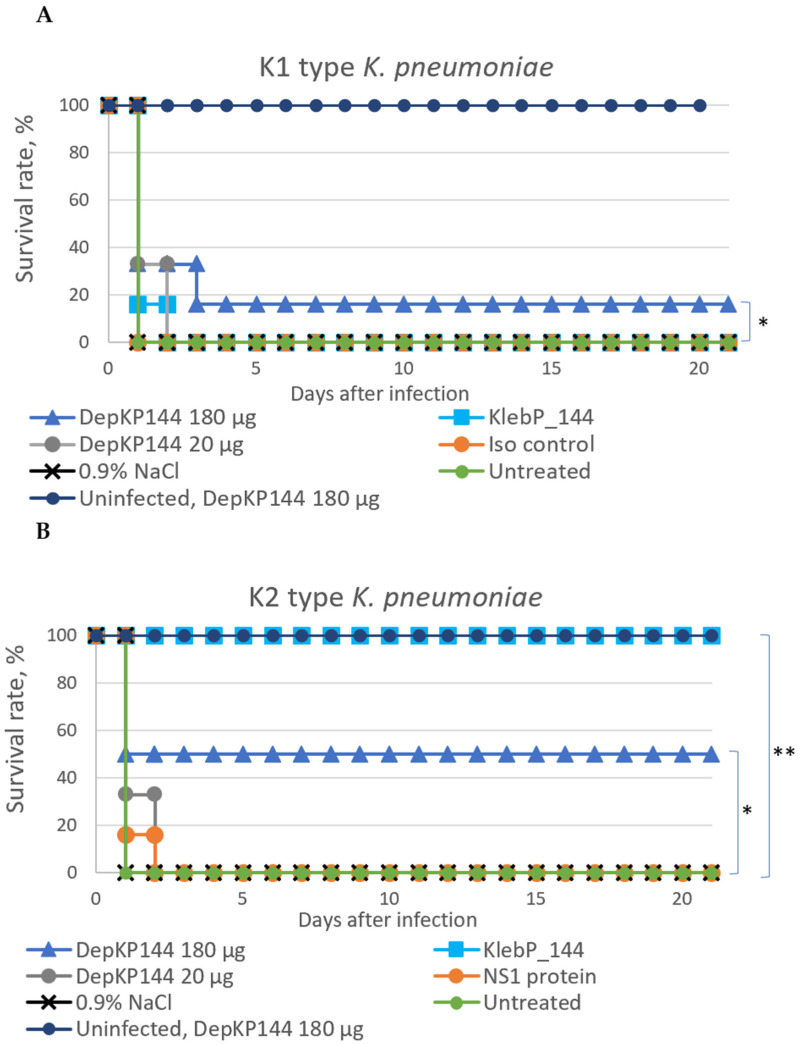
Efficacy of DepKP144 in post-exposure prophylaxis after infection by *K. pneumoniae* strains (**A**) CEMTC 9596 (K1-type) and (**B**) CEMTC 2067 (K2-type). Female BALB/c mice (18 g) were treated i.p. with DepKP144, bacteriophage KlebP_144 or NS1 protein at the indicated doses 2 h after injection of 1 × 10^8^ colony-forming units of *K. pneumoniae* strains CEMTC 2067 (K2-type) and CEMTC 9596 (K1-type). * *p* < 0.05 (log-rank test), ** *p* < 0.01 (log-rank test).

Therapeutic outcomes varied considerably depending on the treatment regimen. Mice infected with *K. pneumoniae* CEMTC 9596 (K1-type) and treated with phage KlebP_144, as well as untreated animals infected with the same strain, did not survive. DepKP144 monotherapy provided a limited but measurable survival benefit. At the higher dose of 180 µg per mouse, approximately 17% of animals infected with the K1-type strain survived. Strikingly, reducing the dose to 20 µg per mouse completely abolished this protective effect, resulting in uniform mortality. This dose-dependent loss of activity suggests a narrow therapeutic window for DepKP144 under the tested conditions (Figure 8).

Mice infected with the bacteriophage host strain *K. pneumoniae* CEMTC 2067 (K2-type) and subsequently treated with phage KlebP_144 achieved 100% survival, highlighting the potent lytic activity of the phage in vivo. By contrast, untreated animals infected with the same strain did not survive. DepKP144 monotherapy again conferred a limited survival benefit: at the higher dose of 180 µg per mouse, approximately 50% of animals infected with the K2-type strain survived. However, reducing the dose to 20 µg per mouse completely eliminated this protective effect, leading to uniform mortality. As observed for the K1-type strain, these results indicate a narrow therapeutic index for DepKP144 under the experimental conditions employed (Figure 8).

## 3. Discussion

It is known that depolymerases generally lack strong lytic activity and are incapable of significantly reducing the titer of host bacteria [24]. Most often, depolymerase activity is associated with structural capsid proteins of bacteriophages that mediate receptor binding [26]. Depolymerases typically exhibit a modular architecture consisting of three structural domains: a conserved N-terminal domain, a variable central domain, and a C-terminal domain [24]. Genome analysis of bacteriophage KlebP_144 revealed a gene encoding a fiber protein with putative depolymerase activity. Structural analysis of the deduced amino acid sequence indicated that the central domain of DepKP144 shares similarity with hydrolases, lyases, and pectate lyases. The amino acid sequence of DepKP144 showed high similarity to several fiber proteins, which, according to BLASTp comparison, belong to phages of the *Podoviridae* family. Specifically, high similarity was observed between DepKP144 and the fiber proteins of phages KP11 (coverage 100%, identity 98.60%) and RCIP0048 (coverage 100%, identity 98.54%). None of these homologous depolymerases have been previously produced as recombinant proteins and characterized. The genes of depolymerases structurally similar to DepKP144 were encoded in the genomes of bacteriophages specific to both K2- and K1-type *K. pneumoniae*.

In this study, DepKP144 from phage KlebP_144 was successfully expressed and purified. DepKP144 exhibited specific activity against both K1- and K2-type *K. pneumoniae* cells but showed no specific activity against other K-types tested (K9, K16, K17, K22, K35, K49, K51, K57, K63, and K108). The efficacy of DepKP144 against K1- and K2-type *K. pneumoniae* was further evaluated in vivo and in vitro. Initially, the ability of recombinant DepKP144 to inhibit the growth of planktonic cultures of K1- and K2-type *K. pneumoniae* was assessed. The results demonstrated that DepKP144 exhibits moderate hydrolytic activity, reducing the bacterial titer by approximately one order of magnitude (10-fold). Subsequently, the ability of this depolymerase to affect the viability of *K. pneumoniae* cells within biofilms was investigated. Bacterial biofilms are complex microbial communities adhered to biological or abiotic surfaces and encased within a self-produced extracellular polymeric matrix comprising polysaccharides, extracellular DNA, lipids, and proteins [27]. Through various mechanisms, biofilms protect microorganisms from adverse environmental factors, thereby enhancing their antimicrobial resistance. DepKP144 was shown to inhibit biofilm formation and to disrupt mature biofilms formed by hypermucoviscous K1- and K2-type *K. pneumoniae* strains, even in the absence of antibiotics.

It is important to emphasize that depolymerase does not directly kill target bacteria but merely degrades their capsular polysaccharide (CPS). Phage-encoded depolymerases alone cannot efficiently eradicate bacterial biofilms. A far more potent antibacterial effect is achieved when depolymerases are combined with other antimicrobial agents, such as antibiotics, chemicals or bacteriophages [28,29]. The mechanism of synergy between phage depolymerases and antibiotics, for example, is predicated on the removal of CPS, which loosens the packing of bacteria within the biofilm and thereby facilitates antibiotic penetration into the matrix. Latka and Drulis-Kawa [30] demonstrated that combining depolymerase with ciprofloxacin and phages is effective against biofilms of multidrug-resistant *K. pneumoniae*. Wu et al. [31] showed that phage depolymerase Dep42 enhances the activity of polymyxin against *K. pneumoniae* biofilms. Furthermore, in a study by Yang and colleagues, the combined application of depolymerase Dep37 and kanamycin against carbapenem-resistant *K. pneumoniae* (CRKP) biofilms also produced a synergistic effect, potentiating the bactericidal action of the antibiotic [32]. According to a review by Guo et al. [24], antibiotics such as ciprofloxacin, colistin, and polymyxin exhibit synergy with phage depolymerases, whereas other antibiotics (e.g., imipenem and amikacin) do not yield such an effect. Consequently, successful synergistic degradation of biofilms using depolymerases requires careful selection of the appropriate antibiotic companion.

Notably, depolymerases demonstrate more pronounced antimicrobial activity in vivo, as capsule degradation renders bacterial cells susceptible to the host immune system. Phage-encoded depolymerases have recently been shown to be effective in treating *Galleria mellonella* larvae infected with K3-, K21-, K44-, and K63-type *K. pneumoniae* [33]. Furthermore, phage-derived depolymerases have been reported to reduce infection by K30-type *K. pneumoniae* in a murine aspiration pneumonia model and to protect mice against K1-, KN1-, K47-, K57-, and K64-type *K. pneumoniae* in murine sepsis models [34,35,36,37,38,39]. In contrast, anti-capsular activity was also demonstrated for DepKP144: treatment of mice infected with K1- and K2-type *K. pneumoniae* resulted in survival rates of 17% and 50%, respectively.

In the study by Majkowska-Skrobek et al., the efficacy of several Klebsiella phage-borne depolymerases was evaluated in *Galleria mellonella* and murine models of *K. pneumoniae* infection, showing that depolymerase administration significantly reduced mortality for specific “strain–capsular type” combinations, but with a strongly strain- and serotype-dependent protective effect [40]. Subsequent work demonstrated that the K57-specific depolymerases Dep_kpv79 and Dep_kpv767 provided 80–100% survival in murine models of lethal sepsis and soft-tissue infection when administered as a single dose shortly after infection, with the authors attributing this efficacy to capsule stripping and enhanced susceptibility to complement-mediated killing, whereas delayed administration resulted in reduced survival [41]. The therapeutic potential of depolymerases has also been demonstrated for K1-, K2-, KN1-, KL47-, and K20-specific enzymes, which conferred protection only when the infecting strain expressed the matching capsular type [34,36,39,42,43,44]. Collectively, these data, together with observations on the possible immunomodulatory role of CPS degradation products and the diversity of depolymerase domain architectures, underline the narrow serotype specificity of most enzymes, the critical importance of timing and dosing, and the notion that depolymerases should primarily be viewed as anti-virulence components of combination therapy rather than stand-alone bactericidal agents [40,41]. In this context, our study is, to our knowledge, the first to describe a depolymerase, DepKP144, capable of protecting experimental animals against two distinct K-types of *K. pneumoniae*, which distinguishes it from previously reported strictly serotype-specific depolymerases and suggests that broader or dual capsular specificity, while still rare, is achievable within the structural and functional diversity of *Klebsiella* phage depolymerases.

In conclusion, this study provides the first characterization of DepKP144, a depolymerase capable of inhibiting biofilm formation, disrupting mature biofilms, and protecting model animals against infections caused by K1- and K2-type *K. pneumoniae*. This research has several limitations. The study examined only *K. pneumoniae* isolates collected within the territory of the Russian Federation. Furthermore, additional investigations using animal models, such as a *K. pneumoniae*-induced pneumonia model, are warranted. The combined application of depolymerases with antibiotics was also not explored. Finally, the complex nature of the interactions between phage depolymerases and host bacterial cells cannot be overlooked. A comprehensive understanding of their antibacterial effects requires further experimental efforts aimed at elucidating the detailed underlying mechanisms.

## 4. Materials and Methods

### 4.1. Bacterial Strains, Phage, and Growth Conditions

Clinical isolates of *K. pneumoniae* were provided by the Collection of Extremophilic Microorganisms and Type Cultures (CEMTC; Institute of Chemical Biology and Fundamental Medicine, Siberian Branch of the Russian Academy of Sciences, Novosibirsk, Russia). In total, 19 K1-type and 35 K2-type *K. pneumoniae* isolates, as well as 10 strains representing other capsular types (K9, K16, K17, K22, K35, K49, K51, K57, K63, and K108), were included in this work. Detailed strain information is provided in Supplementary Table S1.

Molecular cloning of the pET28a_depKP144 expression construct was performed in *Escherichia coli* XL1-Blue (*recA1 endA1 gyrA96 thi-1 hsdR17(rᴋ^−^ mᴋ^+^) supE44 relA1 lac [F′ proAB lacIᵟZΔM15 Tn10 (Tetᴿ)]*). For recombinant protein expression, the construct was subsequently transferred into the expression host *E. coli* BL21(DE3) (*F^−^ ompT gal dcm lon hsdSʙ(rʙ^−^ mʙ^−^) λ(DE3 [lacI lacUV5-T7p07 ind1 sam7 nin5]) [malB^+^] K-12 (λS)*). All bacterial strains were cultivated using Lysogeny Broth (LB) medium under standard cultivation conditions.

Bacteriophage *Klebsiella pneumoniae* KlebP_144, isolated and characterized at the Institute of Chemical Biology and Fundamental Medicine (ICBFM SB RAS, Novosibirsk, Russia), was used in this study. The purified Kleb_P144 bacteriophage preparation was kindly provided by Prof. N.V. Tikunova, CEMTC ICBFM SB RAS. The complete genome sequence of this phage is available in the NCBI GenBank database under accession number PV528436.

### 4.2. Animals

Female BALB/c mice were procured from the certified animal facility of the Federal State Research Center of Virology and Biotechnology “Vector” (FSRC VB “Vector”). All procedures involving animals were conducted in strict accordance with the principles of the European Union Directive 2010/63/EU on the protection of animals used for scientific purposes. The study protocol was reviewed and approved by the Institutional Animal Care and Use Committee (IACUC) of the Institute of Cytology and Genetics, Siberian Branch of the Russian Academy of Sciences (Novosibirsk, Russia).

Female BALB/c mice, six weeks of age and weighing 18–22 g, were housed under specific conditions with ad libitum access to food and water. Animals were acclimatized for at least 7 days before infection and were randomly assigned to experimental groups.

### 4.3. Phylogenetic and In Silico Structural Analysis of C-Terminal Domain of Depolymerase DepKP144

Phylogenetic relationships were inferred using the Maximum Likelihood approach implemented in MEGA X, based on aligned nucleotide sequences and applying the Kimura two-parameter substitution model. Tree robustness was assessed by bootstrap analysis, with evolutionary rate heterogeneity among sites modeled using a discrete gamma distribution and a proportion of invariable sites, after excluding poorly aligned positions by partial deletion.

The C-terminal domain of the DepKP144 depolymerase (DepKP144, GenBank accession XRR08247.1) underwent bioinformatic characterization using several complementary platforms: the InterPro software suite (https://www.ebi.ac.uk/interpro/search/sequence/, accessed on 21 January 2026), the HHpred server (https://toolkit.tuebingen.mpg.de/tools/hhpred, accessed on 21 January 2026), and the COFACTOR function prediction tool (https://aideepmed.com/COFACTOR/, accessed on 19 January 2026) [45]. The solubility profile of the DepKP144 protein was assessed in silico using the Protein-sol server (https://protein-sol.manchester.ac.uk, accessed on 15 August 2024) [46] and the SoluProt predictor (https://loschmidt.chemi.muni.cz/soluprot, accessed on 15 January 2026) [47]. Three-dimensional structural predictions for DepKP144 were computed via AlphaFold2 [48] (https://colab.research.google.com/github/sokrypton/ColabFold/blob/main/AlphaFold2.ipynb, accessed on 21 May 2025) and AlphaFold3 (accessed on 19 January 2026) [49]. Molecular visualization and structural rendering were carried out using UCSF Chimera, version 1.15 [50].

### 4.4. Cloning, Expression and Purification

The depKP144 gene was amplified by PCR from KlebP_144 genomic DNA using primers DepKP144_BspI_U (5′-GAAAGCCATGGGAATGTTAGACAGACTGAATCAGCCGAAAG-3′) and DepKP144_BamHI_L (5′-CCCTTGGATCCGTCAAGAAGTTTACGATAACGTCGTCAAGG-3′). The PCR product and the pET28a vector were digested with Bsp19I (an isoschizomer of NcoI) and BamHI (Sibenzyme, Novosibirsk, Russia) and ligated using standard cloning procedures [51]. The resulting construct, pET28a_depKP144, was introduced into *E. coli* XL1-Blue and verified by colony PCR with primers pET-SeqU (5′-TGCTAGTTAGTAGTATTGCTCAGCG-3′) and pET-SeqL (5′-GGTTCTGGTTCTGGTTCTGGTTCTGGCCATA-3′), followed by Sanger sequencing on a 3500 Genetic Analyzer (Thermo Fisher Scientific, Foster City, CA, USA).

For recombinant expression, *E. coli* BL21(DE3) was transformed with pET28a_depKP144 and selected on LB agar containing 25 µg/mL kanamycin. Cultures were grown at 37 °C to OD_600_ = 0.6–0.7, and expression was induced with IPTG over a range of concentrations (10–1000 µM) and temperatures (12, 20, 30, and 37 °C) for 16–18 h with shaking at 180 rpm. Cells were harvested (6000× *g*, 10 min), resuspended in 50 mM Tris-HCl (pH 8.0), and lysed by sonication (Sonopuls HD 2070, Bandelin Electronic GmbH & Co. KG, Berlin, Germany). Expression levels and cellular localization of DepKP144 were assessed by SDS-PAGE.

DepKP144 was recovered from inclusion bodies under denaturing conditions by immobilized metal-affinity chromatography on Ni–NTA agarose (Sigma-Aldrich, St. Louis, MO, USA) equilibrated in 6 M urea, 50 mM NaH_2_PO_4_, 300 mM NaCl, 10 mM imidazole (pH 8.0). Non-specifically bound proteins were removed by sequential washes with 50, 100, 200, and 500 mM imidazole in the same buffer. Refolding was performed by stepwise dialysis at 4 °C against three buffers with decreasing concentrations of urea (1.5 → 0.5 → 0 M) and imidazole (50 → 10 → 0 mM), in 300 mM NaCl and 200 mM sucrose, with 0.1% Triton X-100 included in the first two steps. Refolded protein was analyzed by 12% SDS-PAGE (GelDoc Go, Bio-Rad, Hercules, CA, USA), filter-sterilized (0.22 µm), and stored at 4 °C in 50 mM Tris-HCl, 300 mM NaCl, 200 mM sucrose (pH 7.4). Protein concentrations were measured with a Qubit 4.0 fluorometer (Thermo Fisher Scientific, USA).

### 4.5. Capsule-Degrading Activity Assay In Vitro

For the diffusion assay, overnight cultures of different *Klebsiella* strains were diluted 1:100 in 20 mL of LB containing 1% agar and poured into Petri dishes. After drying, the plates were incubated for 24 h at 37 °C. The next day, small wells were made using a micropipette tip, and 50 μL of DepKP144 solution (300 μg/mL) or 50 μL of DepKP144 solution (60 μg/mL) was added to each well; buffer without protein served as a negative control. The plates were incubated for an additional 24 h at 37 °C, and protein activity was assessed visually by the presence of turbid halos (zones of inhibited visible bacterial growth) around wells containing protein solutions in the absence of such halos around the control wells.

The ability of the DepKP144 depolymerase to lyse planktonic *Klebsiella* cells was evaluated using a colony-forming unit (CFU) reduction assay. Each *Klebsiella* strain was grown to the exponential phase (OD600 = 0.5), after which the bacterial cells were harvested by centrifugation at 4000× *g* for 5 min. Following one wash step, the cell pellets were resuspended in R-buffer (50 mM Tris-HCl, pH 8.0) and adjusted to a final concentration of 10^6^ CFU/mL. DepKP144 was then serially diluted in the same buffer to obtain a range of working concentrations. Untreated cell suspensions (without the enzyme) served as negative controls. For the assay, 100 µL aliquots of the bacterial suspension were mixed with the respective DepKP144 dilutions in a 96-well plate and incubated at 37 °C for 2 h. Subsequently, samples from each well were spotted or spread onto LB agar plates, and viable colonies were enumerated after overnight incubation.

### 4.6. In Vitro Biofilm Assay

The influence of the DepKP144 depolymerase on biofilm development and structural integrity in *K. pneumoniae* was examined in vitro using a crystal violet-based microtiter plate assay [52,53]. The investigation included clinical isolates representing distinct capsular serotypes, specifically strain 2754 (K2) and strain 2894 (K1).

To assess the inhibition of biofilm formation, bacterial cultures were propagated to the exponential growth phase, normalized to an optical density of 0.1 at 600 nm, and subsequently diluted 1:100 in fresh LB. From these suspensions, 180 µL aliquots were distributed into sterile flat-bottom 96-well plates, followed by the addition of 20 µL of DepKP144 prepared in S-buffer to yield final enzyme concentrations of 75 or 300 µg/mL. Control wells were supplemented with an equivalent volume of phosphate-buffered saline (PBS). The plates were then incubated without agitation at 37 °C for 24 h.

For the biofilm disruption experiments, a slightly modified procedure was followed. Exponential-phase cultures were likewise adjusted to OD600 = 0.1, diluted 100-fold, and seeded into microplate wells (200 µL/well). After a 48 h static incubation at 37 °C to permit the establishment of mature biofilms, the planktonic fraction was discarded, and the wells were washed three times with PBS. Next, 200 µL of DepKP144 solution at the specified concentrations, or PBS alone as a control, was introduced, and the plates were incubated for an additional 6 h at 37 °C.

Residual biofilm biomass was quantified by crystal violet staining. Following the respective treatments, wells were rinsed with PBS and allowed to air-dry to fix the adherent biofilms. The fixed biomass was stained with a 0.1% (*w*/*v*) crystal violet solution for 20 min, after which excess stain was removed by three thorough washes with water. Once dried, the retained dye was extracted using 96% ethanol, and the absorbance of the solubilized material was recorded at 570 nm. All assays were performed as a minimum of three independent biological experiments, each incorporating three technical replicates.

### 4.7. Murine Model of Systemic Klebsiella Infection

The therapeutic efficacy of DepKP144 was assessed in vivo using a murine model of disseminated *K. pneumoniae* infection, modified from a previously described procedure [51]. In brief, *K. pneumoniae* strains CEMTC 2067 (K2-type) or CEMTC 9596 (K1-type) were propagated overnight in LB at 37 °C. The bacterial cells were pelleted by centrifugation, rinsed twice with sterile physiological saline (0.9% NaCl), and resuspended to an optical density at 600 nm corresponding to the target inoculum concentration. Six-week-old female BALB/c mice received an intraperitoneal (i.p.) challenge consisting of 0.1 mL of the bacterial suspension, delivering a total of 1 × 10^8^ colony-forming units (CFU) per animal. Seven experimental cohorts (intact controls; uninfected DepKP144 controls; infected untreated; infected + NS1 at 20 or 100 µg; infected + KlebP_144 phage at 10^9^ PFU; infected + DepKP144 at 20 or 180 µg). Six mice were assigned to each experimental and control group (*n* = 6). Mice were randomly assigned to the following treatment groups: (i) intact controls (uninfected and untreated); (ii) protein controls (uninfected, treated with DepKP144); (iii) infection controls (infected, untreated); (iv) negative protein controls (infected, treated with 20 µg or 100 µg of the heterologous protein NS1 per animal); (v) phage-treated group (infected, treated with 10^9^ PFU of bacteriophage KlebP_144 per mouse); and (vi) DepKP144-treated groups (infected, receiving either 20 µg or 180 µg of DepKP144 alone per mouse).

At two hours post-infection, the mice were administered a single i.p. injection (0.5 mL volume) of one of the following treatments: DepKP144 at 180 µg or 20 µg per mouse; wild-type bacteriophage KlebP_144 at a dose of 1 × 10^9^ plaque-forming units (PFU) per mouse; or the unrelated control protein NS1 at 20 µg or 100 µg per mouse [54]. Animals in the control group received an equivalent volume of saline alone. Survival was monitored daily over a 14-day period, serving as the primary endpoint, with body weight recorded at the same intervals as an additional clinical indicator. The heterologous control protein NS1 (non-structural protein 1 of Omsk hemorrhagic fever virus) was produced in-house as described previously in [54]. To confirm bacteremia, peritoneal washings were collected from all deceased animals. The samples were serially diluted in sterile buffer and plated on selective medium to quantify bacterial burden. High titers of *Klebsiella pneumoniae* (>10^6^ cfu/mL) were detected in all dead animals.

### 4.8. Statistical Analysis

Data obtained from serial dilution assays were compared between control and treatment groups using one-way analysis of variance (ANOVA). A threshold of *p* < 0.01 was adopted to define statistically significant differences in bacterial viability across groups.

For the in vivo murine experiments, survival curves were generated and compared by means of the log-rank (Mantel–Cox) test. Statistical significance was accepted at probability levels of *p* < 0.05 or *p* < 0.01. All computational analyses, including both ANOVA and log-rank testing, were carried out using GraphPad Prism software, version 8.0 (GraphPad Software Inc., San Diego, CA, USA).

## Data Availability

Data are contained within the article and Supplementary Materials.

## Supplementary Materials

The following supporting information can be downloaded at: https://www.mdpi.com/article/10.3390/ijms27125466/s1.

## Abbreviations

The following abbreviations are used in this manuscript:

CRKP	Carbapenem-resistant *K. pneumoniae*
K-types	Capsular types
LB	Luria–Bertani
CFU	Colony-forming units
EPS	Exopolysaccharide
LD50	Median lethal dose
CPS	Capsular polysaccharide
SDS-PAGE	Sodium dodecyl sulfate–polyacrylamide gel electrophoresis
PFU	Plaque-forming units
OD	Optical density
PCR	Polymerase chain reaction
IPTG	Isopropyl β-D-1-thiogalactopyranoside
EDTA	Ethylenediaminetetraacetic acid
ANOVA	Analysis of variance
NCBI	National Center for Biotechnology Information
PDB	Protein Data Bank
ICBFM SB RAS	Institute of Chemical Biology and Fundamental Medicine, Siberian Branch of the Russian Academy of Sciences
MDR	Multidrug resistance
R	Resistance
S	Sensitive
XDR	Extensively Drug-Resistant
