# Supplementary material for: Phage-Encoded Depolymerase DepKP144 with Therapeutic Potential Against Both K1- and K2-Type Klebsiella pneumoniae
Source: Int J Mol Sci. 2026 Jun 17;27(12):5466. doi: 10.3390/ijms27125466 (PMC13300036; doi:10.3390/ijms27125466)
Supplement: Supplementary file 1 [file ijms-27-05466-s001.zip › Fig s1 s2.pdf]

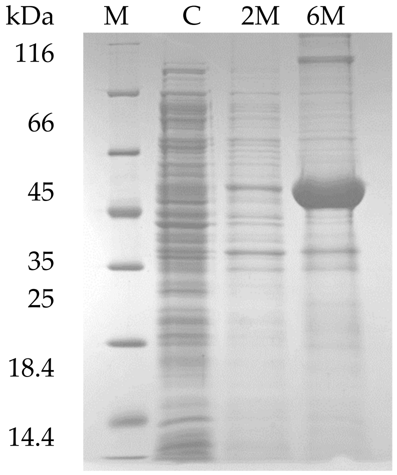

Figure S1. SDS-PAGE of lysates of E. coli BL21(DE3)/pET-28_ DepKP144. C – cytoplasmic fractions; 2 M – inclusion body fraction dissolved in in 50 mM Tris-HCl (pH 8.0), containing 2 M urea; 6 M – inclusion body fraction dissolved in 50 mM Tris-HCl (pH 8.0), containing 6 M urea; M – molecular weight protein marker 26610 (14.4–116 kDa) (Thermo Fisher Scientific, MA, USA).

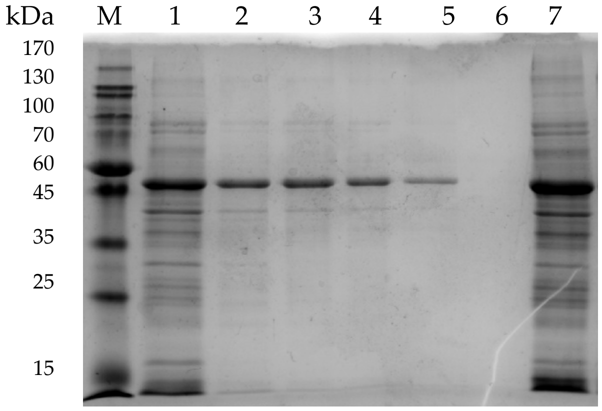

Figure S2. SDS-PAGE of purified pET-28_DepKP144. 1 – elution with wash buffer containing 50 mM imidazole; 2 – elution with wash buffer containing 100 mM imidazole; 3 – elution with wash buffer containing 200 mM imidazole; 4 – elution with wash buffer containing 500 mM imidazole; 5 – elution with wash buffer containing 0.1 M EDTA; 6 - elution with 50 mM NaOH, 1 M NaCl; 7 – cell lysate before loading onto Ni-NTA agarose. M – Protein Ladder RAV11 (Biolabmix, Russia).
