# Supplementary material for: Phage-Encoded Depolymerase DepKP144 with Therapeutic Potential Against Both K1- and K2-Type Klebsiella pneumoniae
Source: Int J Mol Sci. 2026 Jun 17;27(12):5466. doi: 10.3390/ijms27125466 (PMC13300036; doi:10.3390/ijms27125466)
Supplement: Supplementary file 1 [file ijms-27-05466-s001.zip › Table S2.pdf]

Table S2. Comparison of the lytic activity of DepKP144 and phage Kleb_P144.

| CEMTC | DepKP144 | Bacteriophage Kleb_P144 |
|---|---|---|
| **K1-type *K. pneumoniae*** | | |
| 2405 | + | – |
| 2810 | + | – |
| 2894 | + | – |
| 3632 | + | – |
| 3646 | + | – |
| 3647 | – | – |
| 4160 | + | – |
| 6604 | + | – |
| 8557 | + | – |
| 9080 | + | – |
| 9478 | + | – |
| 9479 | + | – |
| 9596 | + | – |
| 9597 | + | – |
| 10016 | + | – |
| 10160 | + | – |
| 10162 | + | – |
| 10634 | + | – |
| 10637 | + | – |
| K2-type *K. pneumoniae* | | |
| 2067 | + | + |
| 2071 | + | + |
| 2291 | + | + |
| 2548 | + | + |
| 2574 | – | + |
| 2576 | – | + |
| 2728 | – | – |
| 2945 | – | – |
| 2946 | – | – |
| 3339 | + | – |
| 3521 | + | – |
| 3522 | + | + |
| 3729 | + | + |
| 3772 | + | – |
| 4065 | + | + |
| 4069 | + | – |

| CEMTC | DepKP144 | Bacteriophage Kleb_P144 |
|---|---|---|
| **K1-type *K. pneumoniae*** | | |
| 4087 | + | + |
| 4090 | + | + |
| 4117 | + | + |
| 4124 | + | – |
| 4128 | + | – |
| 4162 | + | – |
| 4163 | + | – |
| 4169 | + | – |
| 5232 | + | + |
| 5234 | + | + |
| 6824 | + | – |
| 6846 | + | – |
| 6851 | + | – |
| 9609 | + | – |
| 9874 | + | – |
| 10083 | + | – |
| 10125 | + | – |
| 10126 | + | – |
| K9-type *K. pneumoniae* | | |
| 2826 | – | – |
| K16-type *K. pneumoniae* | | |
| 3113 | – | – |
| K17-type *K. pneumoniae* | | |
| 3838 | – | – |
| K22-type *K. pneumoniae* | | |
| 2573 | – | – |
| K35-type *K. pneumoniae* | | |
| 1751 | – | – |
| K49-type *K. pneumoniae* | | |
| 2394 | – | – |
| K51-type *K. pneumoniae* | | |
| 3442 | – | – |
| K57-type *K. pneumoniae* | | |
| 4194 | – | – |
| K63-type *K. pneumoniae* | | |
| 1609 | – | – |
| K108-type *K. pneumoniae* | | |
| 2646 | – | – |
